# Perspectives on periviable birth from the parents of surviving infants

**DOI:** 10.1136/bmjpo-2026-004630

**Published:** 2026-07-02

**Authors:** Jennifer L H Peterson, Sweatha Anathalingam, Edward D Johnstone, Ajit Mahaveer, Debbie M Smith

**Affiliations:** 1 Neonatal Intensive Care Unit, Manchester University NHS Foundation Trust, Manchester, England, UK; 2 The University of Manchester Faculty of Biology Medicine and Health, Manchester, England, UK; 3 Maternity Services, St Mary’s Maternity Hospital, Manchester University NHS Foundation Trust, Manchester, UK; 4 Neonatal Intensive Care Unit, Manchester University NHS Foundation Trust, Manchester, UK

**Keywords:** Infant, Intensive Care Units, Neonatal, Qualitative research

 Advances in neonatal medicine now make survival possible from 22 weeks of gestation.^1^ However, infants born in the periviable period face high risks of death and long-term disability. Decisions about whether to initiate survival-focused intensive care or provide comfort care are often made in emotionally charged and uncertain circumstances, requiring parents to absorb difficult information while experiencing trauma, fear and loss of control.^2^ Research exploring parental perspectives around periviable birth, particularly the experiences of parents from the United Kingdom, is limited.

Our survey presents the long-term perspectives of parents who have a surviving child born in the periviable period. The survey utilised a mixed-methods approach to gather parental feedback (full survey available in online supplemental material). A parental advisory group with lived experience informed the survey design. The survey received favourable ethics review from the North West–Liverpool Central Research Ethics Committee (22/NW/0052).

The electronic medical record systems of a large tertiary neonatal unit in North West England were searched in February 2022 for infants born at 22+0 to 24+6 weeks of gestation who were alive and at least 2 years of age. This resulted in 157 infants (born between 2011 and 2019) whose parents received the postal survey. Ten parents responded (figure 1a). The survey incorporated a validated parent-completed developmental milestone tool: the Parents’ Evaluation of Developmental Status: Developmental Milestones tool,^3^ the Parental Stress Scale questionnaire^4^ and free-text qualitative questions. Reflexive thematic analysis of the qualitative responses identified four themes^5^ (figure 2).

**Figure 1:**
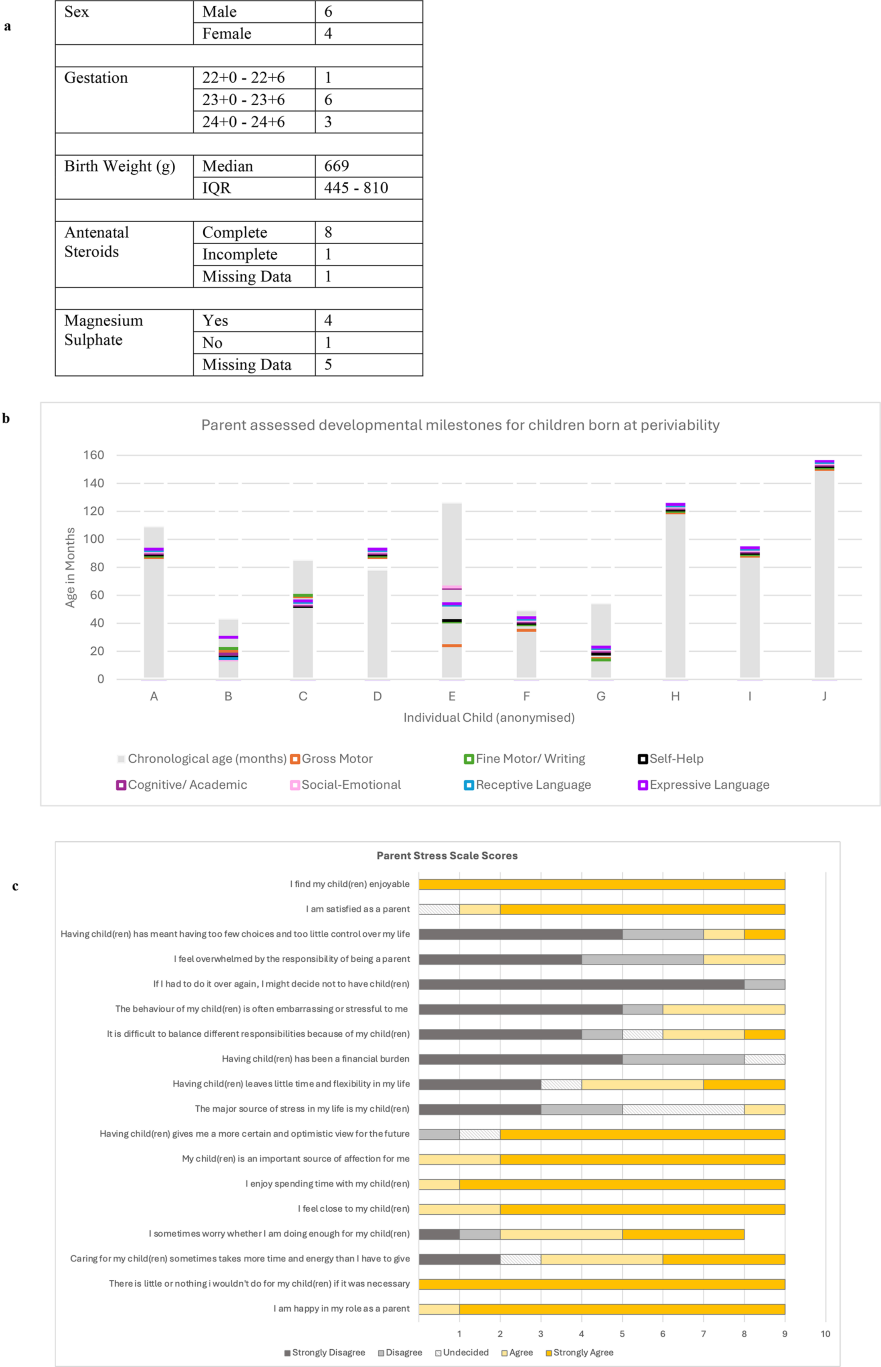
Periviable survivor demographics and outcomes. (a) Demographics; (b) developmental milestones (n=10); (c) parent stress scale responses (n=9; one parent chose not to complete the parental stress scale). (b) Illustrates the child’s corrected chronological age (grey bar) with the highest achieved, parent-reported milestone from the development tool shown with different coloured bars for each developmental domain.

**Figure 2:**
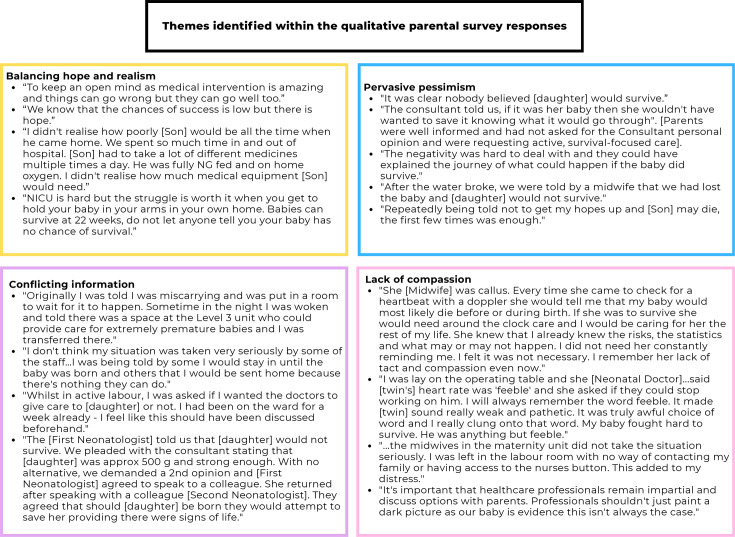
Themes from parent survey.

Developmental outcomes varied across the cohort (figure 1b). Four children were reported to be achieving age-appropriate milestones, while six had varying degrees of developmental delay. Despite this variation, all parents described their children as sources of joy who actively participated in family life, friendships, play and everyday childhood activities. Parental stress scores reflected the significant demands of caregiving, with many reporting that care required more time and energy than they had available (figure 1c). Nevertheless, all expressed satisfaction in their parental role. The qualitative parent responses articulated a nuanced balance of hope and realism throughout their periviable journey, acknowledging the trauma, uncertainty and ongoing challenges, yet emphasising their child’s strengths, individuality and happiness.

In relation to the pre-birth conversations, parents emphasised the pessimism of professionals repeatedly emphasising death and disabilities (figure 2). While parents valued honest communication about relevant risks for their baby, many parents experienced these conversations as disproportionately focused on negative outcomes with limited discussion of even the possibility of survival. Although this survey solely represents the views of parents whose child survived, the survey responses showed that parents wanted balanced conversations that acknowledged the inherent uncertainty within periviable care.

Another theme was the lack of compassion from perinatal professionals. Parents recalled dismissive language, inconsistent information and arbitrary policy-based gestational age thresholds influencing management discussions (figure 2). Some described system-level issues, such as difficulty accessing clinicians with experience of managing periviable infants. These experiences created distrust and had long-lasting emotional impacts. The findings of this survey align with wider reviews, such as the Ockenden report, which highlights deficits in compassionate, trauma-informed practice within maternity care.^6^

This study is limited by its small sample size and potential non-response bias. The response rate may have been influenced by the two-stage, paper-based postal design, which required parents to opt in before receiving the full survey pack. While chosen to maximise inclusivity for families without reliable internet access, this approach may have reduced participation due to the additional steps involved, time burden and/or emotional difficulty of revisiting traumatic experiences. Future research using electronic approaches may improve response rates.

Overall, the findings suggest the need for integration of trauma-informed principles, such as emphasising compassion, provision of balanced, consistent information and respect for parental values, into these pre-birth periviable conversations to improve and support meaningful parental involvement in decision-making.

## Supplementary material

10.1136/bmjpo-2026-004630 online supplemental file 1
