# Supplementary material for: Perspectives on periviable birth from the parents of surviving infants
Source: BMJ Paediatr Open. 2026 Jul 2;10(1):e004630. doi: 10.1136/bmjpo-2026-004630 (PMC13331051; doi:10.1136/bmjpo-2026-004630)
Supplement: online supplemental file 1 [file bmjpo-10-1-s001.pdf]

Participant ID Code:

Manchester University
NHS Foundation Trust

PeriviAble DeLiveries:
ALIgning PArental aNd PhysiCian PrioritiEs (ALLIANCE)

Please feel free to complete this survey in whichever way suits you best. Some parents may choose to complete the survey together; in other cases, parents may wish to complete one survey each.

Completed by (please circle as appropriate): Mother/ Father/ Both Parents

Part 1: Childhood Development Checklist[i] (Appendix A)

There are 7 different development domains listed across the top of Table A (Gross Motor, Fine Motor. Self-Help. Cognitive, Social, Receptive Language and Expressive Language). For each domain, please read the descriptions, starting at the top of page one and working down to circle the one furthest down the list that your child is able to do. The list starts at the top of page one with skills expected for a baby and as the pages progress shows skills that children develop through childhood.

You may find that your child is able to do a development skill that is more advanced in one domain than another. Every child is different and will find certain things easier and others more difficult.

Participant ID Code:

From *Parents' Evaluation of Developmental Status: Developmental Milestones (PEDS:DM)*, Glascoe FP, Robertshaw NS. **www.pedstest.com**
You may reproduce this page.

[redacted]	Gross Motor	Fine Motor/ Writing	Self-Help	Cognitive/Academic	Social/Emo-tional	Receptive Language	Expressive Language
[redacted]	Turns head in supine<br>Chin up in prone	Hands fisted near face	Sucks well	Gazes at black-white objects<br>Follows face	Discriminates mother voice<br>Cries out of distress	Startles to loud noise	Makes sounds other than crying
[redacted]	Chest up in prone<br>Tries to steady head briefly when held	Hands unfisted 50%<br>Retains rattle if placed in hand<br>Holds hands together	Opens mouth at sight of breast or bottle	Follows large highly contrasting objects<br>Recognizes mother	Reciprocal smiling - responds to adult voice & smile	Alerts to voice / sound	Coos (e.g., "oooh" and "aah")<br>Social smile (6 wks)<br>Gurgles when vocalizing
[redacted]	Props on forearms in prone<br>Rolls to side	Hands unfisted 50%<br>Inspects fingers<br>Bats at objects	Brings hands to mouth	Reaches for parent's face<br>Follows objects moved in circle (when in supine)<br>Regards toys	Expression of disgust (sour taste, loud sound)<br>Visually follows person who is moving across a room	Regards speaker	Chuckles<br>Vocalizes when talked to
[redacted]	Sits with trunk support<br>No head lag when pulled to sit<br>Props on wrists<br>Rolls front to back	Clutches at clothes<br>Reaches persistently<br>Plays with rattle<br>Holds hands predominately open	Briefly holds onto breast or bottle	Mouths objects<br>Stares longer at novel faces than familiar ones<br>Shakes rattle<br>Reaches for ring/rattle	Smiles spontaneously at pleasurable sight/sound<br>Stops crying at parent voice<br>To and fro alternating vocalizations	Orients head in direction of a voice<br>Stops crying to soothing voice	Laughs out loud<br>Vocalizes when alone
[redacted]	Sits with pelvic support<br>Rolls back to front<br>Parachute sits with arms supporting trunk (anterior protection)	Grasps cube using whole hand (palmer grasp)<br>Transfers objects: hand-mouth-hand<br>Holds hands together<br>Reaches/grasps dangling ring	Gums/mouths pureed food	Turns head to look for dropped spoon<br>Regards pellet or small cracker	Recognizes caregiver visually<br>Forms attachment -relationship to caregiver	Begins to respond to name	Says "Ah-goo" (or other vowel +consonant combinations)<br>Razz, squeal<br>Expresses anger with sounds other than crying

[illegible]

From *Parents' Evaluation of Developmental Status: Developmental Milestones (PEDS:DM)*, Glascoe FP, Robertshaw NS. **www.pedstest**
You may reproduce this page.

[redacted]	Gross Motor	Fine Motor/ Writing	Self-Help	Cognitive/Academic	Social/Emo-tional	Receptive Language	Expressive Language
[redacted]	Sits momentarily propped on hands<br>Pivots in prone (on belly)<br>Prone--bears weight on one hand	Transfers hand-hand<br>Rakes pellet<br>Takes second cube - holds on to one<br>Reaches with one hand	Feeds self crackers<br>Places hands on bottle	Touches reflection and vocalizes<br>Removes cloth on face<br>Bangs & shakes toys	Stranger anxiety: recognizes familiar vs. unfamiliar people	Stops momentarily to "no"<br>Gestures for "up"	Reduplicate babble with consonants<br>Listens then vocalizes when adult stops<br>Smiles/Vocalizes to mirror
[redacted]	Bounces when held<br>Sits w/o support--Steady<br>Puts arms out to sides for balance (Lateral protection)	Grasps using side of hand (radial-palmar grasp)	Refuses excess food	Explores different aspects of a toy<br>Observes cube in each hand<br>Finds partially hidden object	Looks from object to parent and back when wanting help (e.g., with a wind-up toy)	Looks toward familiar object when named<br>Attends to music	Increasing variety of syllables<br>Repeats same syllables (e.g., "mamamama")
[redacted]	Gets into sitting<br>Commando crawls<br>Pulls to sitting/ kneeling	Bangs spoon after a demo<br>Grasps with all four fingers and side of thumb (Scissor grasp)<br>Takes cube out of cup<br>Pulls large peg out	Holds own bottle<br>Finger feeds Cheerios or string beans	Seeks object after it falls silently to the floor	Lets parents know when happy vs. upset<br>Engages in gaze monitoring:adult looks away and child follows adult glance with own eyes	Responds to "come here"<br>Looks for family members when asked, "Where's Mama?...etc.	Says "Mama" (non-specific)<br>Non-reduplicate babble (e.g., "wapa")<br>Imitates sounds
[redacted]	"Stands" on feet and hands<br>Begins creeping<br>Pulls to stand<br>Crawls with all four limbs straightend (Bear walks)	Grasps with two finger and thumb below (Radial-digital)<br>Bangs 2 cubes together	Bites, chews cookie	Inspects parts of a bell<br>Rings bell after demo<br>Pulls string to obtain an attached toy out of reach	Uses sounds to get attention<br>Separation anxiety<br>Follows a point "Oh look at... "<br>Recognizes familiar people visually	Enjoys gesture games<br>Orients to name well<br>Turns head upward and diagonally to view source of sound	"Mama" (non-specific)<br>Increased non-reduplicate babble (e.g, "wapa" +"mada")<br>Imitates sounds<br>Gestures (e.g., reaching, pointing)

Participant ID Code:

From *Parents' Evaluation of Developmental Status: Developmental Milestones (PEDS:DM),* Glascoe FP, Robertshaw NS. **www.pedstest.co**
You may reproduce this page.

	Gross Motor	Fine Motor/ Writing	Self-Help	Cognitive/Academic	Social/Emo-tional	Receptive Language	Expressive Language
[redacted]	Creeps well<br>Cruises around furniture- two hands<br>Stands--one hand held<br>Walks--two hands held	Clumsy release of cube<br>Grasps pellet with side of index finger and thumb (inferior pincer grasp)<br>Isolates index finger and pokes	Drinks (not sucks) from cup held for him/her	Uncovers toy under cloth<br>Pokes at pellet in bottle<br>Tries to put cube in cup, but may not be able to let go	Experiences fear<br>Looks preferen-tially when name is called	Enjoys Peek-A-Boo<br>Waves "bye-bye" back	Says "Dada" (specific)<br>Waves bye-bye<br>Adds new consonant sounds to babbling
	Walks--one hand held<br>Pivots in sitting<br>Cruises furniture holding on with one hand<br>Stands for a few seconds	Throws objects<br>Stirs with spoon	Cooperates in dressing	Finds toy under cup<br>Looks at pictures in book	Gives objects to adult for action after demonstra-tion (lets adult know he needs help)	Stops activity when told "no"<br>Bounces to music	Says first word<br>Vocalizes to songs
	Stands well with arms high and legs splayed (Posterior protection)<br>Independent steps	Scribbles after demo<br>Fine pincer grasp of pellet<br>Holds crayon<br>Attempts tower of two cubes	Cooperates in dressing<br>Finger feeds part of meal<br>Takes hat off	Rattles spoon in cup<br>Lifts box lid to find toy	Shows objects to parent to share interest<br>Points in order to get desired object (Proto-imperative pointing)	Follows 1-step command with gesture<br>Recognizes names of two objects–looks at each correctly when named	Points in order to get de-sired object (Proto-impera-tive pointing)<br>Uses several gestures with vocalizing (waving, reach-ing, etc.)
	Walks with arms high and out (high guard)	Attempts to release pellet in bottle	Drinks from cup wtih some spilling	Dangles ring by string<br>Reaches around clear barrier to obtain object<br>Unwraps toy in cloth	Shows desire to please caregiver<br>Solitary play<br>Functional play	Looks appropriately when asked<br>"Where's the ball?", etc.	Uses three words<br>Immature jargoning (Inflec-tions without real words)
	Stands without pull-ing up<br>Falls by collapse<br>Walks well	Attempts to release pellet in bottle<br>Imitates back--forth scribble<br>Adds third cube to a two cube tower<br>Puts round peg in & out of hole	Removes socks/ shoes<br>Chews well<br>Puts spoon in mouth typically turning it over	Dumps pellet out of bottle after demo	Points at object to express inter-est (e.g., to get parent to name it) (Proto-declarative pointing)<br>Purposeful ex-ploration of toys through trial and error	Follows one-step command without gesture (e.g., "give it")	Names one object<br>Points at object to express interest (e.g., to get parent to name it) (Proto-declara-tive pointing)

From *Parents' Evaluation of Developmental Status: Developmental Milestones (PEDS:DM),* Glascoe FP, Robertshaw NS. **www.pedstest.co**
You may reproduce this page.

	Gross Motor	Fine Motor/ Writing	Self-Help	Cognitive/Academic	Social/Emo-tional	Receptive Language	Expressive Language
[redacted]	Stoops to pick up toy<br>Creeps up stairs<br>Runs stiff-legged<br>Walks carrying toy<br>Climbs on furniture	buildds 3-4 cube tower<br>Place 10 cubes in cup<br>Releases pellet into bottle	Uses spoon--some spill<br>Attempts to brush own hair<br>Fusses to be changed	Turns pages in book<br>Places circle in single shape puzzle<br>● △ □	Shows empathy (someone else cries child looks sad)<br>Hugs adult in reciprocation<br>Recognizes with-out a demo that a toy requires acti-vation and hands it to an adult if can't operate	Points to 1 body part<br>Points to 1 object of 3<br>Gets object from another room upon demand	Uses 3 - 5 words<br>Mature jargoning with real words
	Stands on one foot with slight support<br>Walks backwards<br>Walks up stairs - one hand held	Puts several round pegs in board (with urging)<br>Scribbles spontane-ously	Picks up and drinks from cup<br>Fetches and carries objects (same room)	Dumps pellet out without demo<br>Places circle in formboard<br>Finds toy observed to be hidden under layers of covers	Kisses by touch-ing lips to skin<br>Periodically visu-ally relocates caregiver<br>Self-conscious: embarrassed when aware of people observing	Understands simple com-mands "Bring to mommy"<br>Points to one picture when named	Uses 5-10 words
	Creeps down stairs<br>Runs well<br>Seats self in small chair<br>Throws ball—stand-ing	Makes 4 cube tower<br>Crudely imitates verti-cal stroke	Removes garment<br>Gets onto adult chair unaided<br>Moves about house without adult	Matches pairs of objects<br>Re-places circle in formboard after it has been turned around (usually with trial and error)	Passes M-CHAT<br>Engages in pretend play with other people (e.g. tea party, birthday party<br>Begins to show shame (when does wrong) & possessiveness	Points to 2 of 3 objects when named<br>Points to 3 body parts<br>Points to self<br>Understands "mine"<br>Points to familiar people when named	Uses 10-25 words<br>Uses giant words (all gone, stop that)<br>Imitates environmental sounds (e.g., animals)<br>Names one picture on demand
	Squats in play<br>Carries large object<br>Goes up stairs held by one hand	Completes round peg board without urging<br>Makes 5-6 cube tower<br>Completes square peg board	Places only edibles in mouth<br>Feeds self with spoon --entire meal	Returns to search for object hidden under cloth after not finding it an adult's closed fists<br>Places square in form board<br>● △ ■	Begins to have thoughts about feelings<br>Engages in tea party with stuffed animals or dolls<br>Kisses with pucker	Points to three pictures<br>Begins to understand her / him / me	Holophrases ("Mommy?"and points at keys, meaning "These are Mommy's keys")<br>Two-word combinations<br>Answers requests wtih "no"

Participant ID Code:

From *Parents' Evaluation of Developmental Status: Developmental Milestones (PEDS:DM)*, Glascoe FP, Robertshaw NS. www.p
You may reproduce this page.

[redacted]	Gross Motor	Fine Motor/ Writing	Self-Help	Cognitive/Academic	Social/Emo-tional	Receptive Language	Expressive Language
[redacted]	Goes up stairs holding rail, putting both feet on each step<br>Kicks ball wtih demo<br>Walks with one foot on walking board	Closes box with lid<br>Imitates vertical line<br>Imitates circular scribble	Uses spoon well<br>Drinks from cup well<br>Unzips zippers<br>Puts shoes on partway	Completes formboard with three shapes<br>● ▲ ■	Watches other children intensely<br>Begins to show defiant behavior	Points to 4 – 5 pictures when-named<br>Points to 5 – 6 body parts<br>Points to 4 pieces of clothing when named	Uses 25-50 words<br>Asks for more<br>Adds 1-2 words per week
[redacted]	Walks down stairs holding rail, both feet on each step<br>Kicks ball without demo<br>Throws overhand	Makes a single-line "train" of cubes<br>Imitates circle<br>Imitates horizontal line	Opens door using knob<br>Sucks through straw<br>Takes off clothes without buttons<br>Pulls off pants	Sorts objects<br>Matches objects to pictures<br>Shows use of familiar objects	Parallel play<br>Begins to mask emotions for social etiquette	Follows two-step command<br>Understands me / you<br>Points to 5-10 pictures<br>2 word sentences<br>(noun+verb)<br>Telegraphic speech<br>50% intelligibility<br>Refers to self by name<br>Names 3 pictures	Uses two-word sentences (noun+verb)<br>Telegraphic speech<br>50 + words in vocabulary<br>50% intelligibility<br>Refers to self by name<br>Names 3 pictures<br>Adds new consonant sounds (e.g., "g", "h")
[redacted]	Jumps from bot-tom step, one foot leading<br>Walks on toes after demo<br>Walks backward 10 steps	Strings large beads (awkwardly)<br>Unscrews jar lid<br>Turns paper pages (often several at once)	Holds self and/or verbalizes toilet needs<br>Pulls pants up with assistance	Matches shapes<br>Matches colors	Reduction in separation anxiety	Understands "just one"	Repeats 2 digits<br>Begins to use pronouns<br>(I, me, you)<br>Names 10-15 pictures
[redacted]	Goes up stairs with rail, alternating feet<br>Jumps in place<br>Stands with both feet on balance beam<br>Walks with one foot on balance beam	Makes an 8 cube tower<br>Makes "a train" of cubes and includes a stack<br>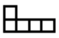	Washes hands<br>Puts things away<br>Brush teeth with assistance	Re-places circle in formboard after it has been turned around (little or no trial and error)<br>Points to small details in pictures	Imitates adult activities (e.g., sweeping, talking on phone, pre-tending to hunt animals)	Follows 2 prepositions: "Put block in . . .on box"<br>Points to objects by use: "ride in" ... "put on feet" . . ."write with"	Echolalia and jargoning gone<br>Names objects by use<br>Refers to self with correct pronoun<br>Recites parts of well-known story/fills in words

From *Parents' Evaluation of Developmental Status: Developmental Milestones (PEDS:DM)*, Glascoe FP, Robertshaw NS. www.p
You may reproduce this page.

[redacted]	Gross Motor	Fine Motor/ Writing	Self-Help	Cognitive/Academic	Social/Emo-tional	Receptive Language	Expressive Language
[redacted]	Walks swinging arms opposite of legs (synchronous gait)	Makes 9-10 cube tower<br>Puts 6 square pegs in pegboard<br>Imitates cross	Toilet trained<br>Puts on coat unas-sisted	Points to self in photos<br>Points to body parts according to function ("what do you hear with...?")	Begins to take turns<br>Tries to help with household tasks	Understands three prepositions<br>Understands dirty, wet	Gives first and last name<br>Counts to 3<br>Begins to use past tense<br>Enjoys being read to (short books)
[redacted]	Balances on one foot for 3 seconds<br>Goes up stairs, alternating feet, no rail<br>Pedals tricycle<br>Walks heel to toe<br>Catches ball—arms stif	Copies circle<br>Cuts with scissors: side to side (awk-wardly)<br>Imitates bridge of cubes<br>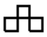<br>Strings small beads well	Eats independently<br>Pours liquid<br>Puts on shoes without laces<br>Spreads with knife<br>Unbuttons	Draws a 2 – 3 part person<br>Understands big/small, more/less<br>Knows own gender<br>Knows own age<br>Matches letters/numerals	Starts to share with/without prompt<br>Fears imaginary things<br>Imaginative play<br>Uses words to describe what someone else is thinking ("Mom thought I was asleep")	Points to parts of pictures (nose of cow, door of car)<br>Understands action words: "playing...washing...blowing"<br>Names body parts when func-tions described<br>Understands negatives<br>Groups objects (foods, toys)<br>Understands long/short	Uses 200+ words<br>3 word sentences<br>Uses pronouns correctly<br>75% intelligibility<br>Acquires new consonant sounds (e.g., "f", "t", "j", "s")<br>Uses plurals<br>Names body parts by use<br>Asks to be read to
[redacted]	Balances on one foot 4-8 sec<br>Hops on one foor 2-3 times<br>Standing broad jump:1-2 ft<br>Gallops<br>Throws ball over-hand 10 ft<br>Catches bounced ball	Copies square<br>Imitates making a complex gate with cubes<br>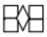<br>Ties single knot<br>Cuts 5 inch circle<br>Uses tongs to transfer<br>Writes part of first-name<br>Works from left to right, top to bottom	Goes to toilet alone<br>Wipes after BM<br>Washes face / hands<br>Brushes teeth alone<br>Buttons<br>Uses fork well	Draws a 4 – 6 part person<br>Can give amounts (usually less than 5) correctly<br>Completes simple analogies: (e.g., dad /boy: mother/ ???, Ice /cold: fire / ???, Ceiling /up: floor/ ???<br>Points to 5 - 6 colors<br>Points to letters/numerals when named<br>Rote counts to 4<br>"Reads" several common signs/store names	Deception – interested in "tricking" others, and concerned about being tricked by others<br>Has a preferred friend<br>Labels happiness, sadness, fear and anger in self<br>Group play	Follows three step commands<br>Points to things that are the same versus different<br>Names things when actions are describe (e.g., it swims in water, you cut with it, it's something you read, it tells time..)	Repeats 4 – 6 syllable sentence<br>Uses 300-1000 words<br>Tells stories<br>May repeat words in sen-tences (e.g., ("Mom, Mom, ... I, I want....")<br>100% intelligibility with few articulation errors<br>Uses "feeling" words<br>Uses words that tell about time

Participant ID Code:

[redacted]	Gross Motor	Fine Motor/ Writing	Self-Help	Cognitive/Academic	Social/Emo-tional	Receptive Language	Expressive Language
[redacted]	Walks down stairs, alternating feet, without using rail<br>Balances on one foot for >8 sec<br>Hops on one foot 15 feet<br>Skips<br>Running broad jump 2-3 ft<br>Walks backward heel-toe<br>Jumps backward	Copies triangle<br>Builds stairs from-model<br>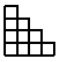<br>Puts paper clip on paper<br>Can use clothespins to transfer small objects<br>Cuts with scissors<br>Writes first name	Spreads with knife<br>Independent dress-ing<br>Bathes indepen-dently	Draws an 8 – 10 part person<br>Gives amounts (< 10)<br>Identifies coins<br>Names letters/numerals out of order<br>Rote counts to 10<br>Names 10 colors<br>Uses letter names as sounds to invent spelling (e.g. NDN)<br>By end of kindergarten:Knows sounds of lconsonants and "short vowels"<br>Reads 25 words	Has a group of friends<br>Apologizes for mistakes<br>Responds verbally to good fortune of others	Knows right and left on self<br>Points to different one in a series<br>Understands "er" endings, (e.g.,(batter, skater).<br>Understands adjectives:bushy, long, thin, pointed<br>Enjoys rhyming words and alliterations<br>Produces words that rhyme<br>Points correctly to "side", "middle", "corner"	Repeats 6 – 8 syllable sentence<br>Defines simple words<br>2000 words<br>Knows telephone number<br>Responds to why questions<br>Retells stories with clear beginning, middle, end<br>Word repetitions in sen-tences wane (single sound repetitions e.g., s-s-s-s-, absent)
[redacted]	Tandem walks<br>Skips	Builds stairs from memory<br>Draws diamond<br>Copies flag<br>Writes first and last name<br>Creates and writes short sentences<br>Forms letters with down-going and counterclockwise strokes	Ties shoes<br>Combs hair<br>Looks both ways at street<br>Remembers to bring belongings	Draws a 12-14 part person<br>Number concepts to 20<br>Simple addition/subtraction<br>Understands seasons<br>Sounds out regularly spelled words<br>Reads (by end of first grade) 250 words	Has best friend of same sex<br>Plays board games<br>Distinguishes fantasy from reality<br>Wants to be like friends and please them<br>Enjoys school	Asks what unfamiliar words mean<br>Can tell which words do not belong in a group	Repeats 8 – 10 word Sentences<br>Describes events in an orderly way<br>Masters sounds of "r" and "l"<br>Knows days of the week<br>10,000 word vocabulary<br>Masters pronunciation of consonant digraphs (e.g., "st-", "dr")
[redacted]	Rides bicycle inde-pendently<br>Bats ball placed on cone<br>Does somersaults	Writing rate increases<br>Stays on line when writing<br>Spaces between words<br>Size of letters be-comes uniform<br>Letter reversals disap-pear	Sticks with tasks (with TV off) for up to 20 minutes<br>Pays attention to teacher when in a group<br>Completes home-work on own<br>Answers and delivers phone messages<br>Completes house-hold chores (with reminders)	Knows sounds of consonant di-graphs (e.g., "ch", "sh")<br>Knows sounds of voweldipthongs (e.g., "oo", "ou")<br>Reads words with-r-controlled vow-els (e.g., "bird", "burn"<br>Starts "reading to learn" not just "learning to read"<br>Two-place addition and subtraction<br>Enjoys reading independently<br>Remembers spelling words	Avoids hurting others in play<br>Learns from mistakes<br>Helps younger children<br>Strong notions about what is fair<br>Takes turns in conversations<br>Delays gratifica-tion and waits to take turn<br>Interested in the opinions of peers	Understands "opposites" and word analogies<br>Answers "who", "why", "when','where" and "how" questions<br>Knows right from left on others<br>Understands days and months	Masters "r" sound in speech<br>Tells time<br>Uses complex and com-pound sentences<br>Talks about a range of topics

From *Parents' Evaluation of Developmental Status: Developmental Milestones (PEDS:DM)*, Glascoe FP, Robertshaw NS. **www.pedstest.com**
You may reproduce this page.

[illegible]

Participant ID Code:

Free-text section: We would like to know more about how your child is growing and developing. If you're happy to please answer the following questions, including as much detail as possible.

- How would you describe your child?
- What does your child enjoy doing?
- What are their strengths?
- What do they find difficult?
- Is there anything that they are unable to or struggle to do compared to their siblings at that age or their peers? If so, what things do they struggle more with?
- Does your child access any additional services, for example, occupational health, speech and language therapy, physiotherapy?
- What would you say are your child's medical issues or concerns?
- Does your child attend nursery or school?
- Do they receive additional help/support at nursery or school?

Participant ID Code:

Part 2: Parental Stress Scale[ii]

This questionnaire asks about the impact that raising a child born extremely premature has had on your experience of parenthood. Please answer the following questions in relation to your experience as a parent specifically with your extremely preterm child. Your answers are confidential.

Parental Stress Scale (this was provided in print here for parents participating the study). The scale can be viewed here: https://www.corc.uk.net/media/2764/parent-stress-scale-fillable-pdf.pdf

Participant ID Code:

Part 3: Free-text Questions - Your Reflections on your pre-birth conversations with healthcare professionals

Thinking back to when you were in extremely preterm labour before you gave birth, can you tell us about your experiences of the following:

- Were you able to discuss what would happen after giving birth with any midwives?
- What was discussed in that conversation?
- Were you able to discuss what would happen after giving birth with any doctors?
- Do you remember if the doctor was an obstetrician (doctor looking after the mother) or a neonatologist (doctors looking after the baby)? Or did you speak with both?
- What was discussed in that conversation?
- What did the healthcare team explain about the implications of being born extremely prematurely for your baby?
- Did the conversation prepare you for what actually happened after you gave birth?
- Did the conversation prepare you for what actually happened during your neonatal stay?
- Did the conversation before birth discuss anything about your baby surviving to going home?
- Did the conversation prepare you for what actually happened after you were discharge home with your baby?
- With the knowledge that you have now about having and raising an extremely preterm child, what would you recommend that doctors discuss with future parents who are in extremely preterm labour?
- Is there anything that you think should not be discussed?
- What would you say to future parents if they were in the same circumstances as you were?

---

[i] https://depts.washington.edu/dbpeds/Screening%20Tools/MilestonesChart_1mo-to-8yr(CCD.2012).pdf from https://depts.washington.edu/dbpeds/Screening%20Tools/ScreeningTools.html

[ii] https://www.corc.uk.net/outcome-experience-measures/parental-stress-scale-pss/. Accessed via this article: https://pubmed.ncbi.nlm.nih.gov/21497029/
