# Supplementary material for: Perspectives on periviable birth from the parents of surviving infants
Source: BMJ Paediatr Open. 2026 Jul 2;10(1):e004630. doi: 10.1136/bmjpo-2026-004630 (PMC13331051; doi:10.1136/bmjpo-2026-004630)
Supplement: Supplementary file 2 [file bmjpo-2026-004630.reviewer_comments.pdf]

PEER REVIEW HISTORY

BMJ Paediatrics Open publishes all reviews undertaken for accepted manuscripts. Reviewers are asked to complete a checklist review form and are provided with free text boxes to elaborate on their assessment. These free text comments are reproduced below.

ARTICLE DETAILS

**TITLE (PROVISIONAL)**	Perspectives on periviable birth from the parents of surviving infants
**AUTHORS**	Peterson, Jennifer LH<br>Ananthalingam, Sweatha<br>Johnstone, Edward D<br>Mahaveer, Ajit<br>Smith, Debbie M

VERSION 1 – REVIEW

**REVIEWER NAME**	Murphy, Claire
**REVIEWER AFFILIATION**	Chelsea and Westminster Hospital NHS Foundation Trust, Department of Neonatology
**REVIEWER CONFLICT OF INTEREST**	No Conflict of Interest
**AI DISCLOSURE**	No, I have not used AI
**DATE REVIEW RETURNED**	16-Mar-2026

**GENERAL COMMENTS**	This is a research letter exploring parental attitudes towards periviable birth.<br>This is an important area to study and the parental voice is essential to consider.<br><br>Comments<br>1. Very small study - 10 respondents (6% of those surveyed) limits the generalisability of the findings and there is ahigh risk of non-response bias.<br>2. Figure 1 a- I think this would be easier to follow in a two-column table<br>3. Figure 1b- This needs greater explanation- the name of the tool should be described and how the results are to be interpreted- e.g. the chronological age of the child and the corresponding age of the most advanced milestone achieved in each domain?<br>4. Figure 1c- I like how this is visually presented<br>5. Limitations- in the discussion of Figure 2, it is important to acknowledge that there was inherent selection bias in this study- parents of periviable infants who died before two years of age were not included. At the most preterm gestation (22 weeks), a majority of infants delivered will die and this group are not represented here. This is clear in the title of the study. However, it should be mentioned in the discussion of the thematic analysis of "pervasive pessimism" and "balancing hope and realism" as it is only survivors being interviewed, even though they may represent only a minority of cases counselled at periviable gestations.

VERSION 1 – AUTHOR RESPONSE

Response to reviewer comments
Thank you for your considered review of our article. We have reflected on your feedback and amended our manuscript accordingly. We hope you enjoy the revised submission.

Reviewer comment: This is a research letter exploring parental attitudes towards periviable birth. This is an important area to study and the parental voice is essential to consider.
Response: Thank you.

Reviewer comment: Very small study - 10 respondents (6% of those surveyed) limits the generalisability of the findings and there is a high risk of non-response bias.
Response: Agreed. We have been transparent about this both in the third paragraph where we outline how the study was conducted and the penultimate paragraph where we highlight the limitations and potential for non-response bias. Despite these limitations, we hope that our article can be considered for publication so that the views of this specific cohort of parents can be present and contribute to the literature and discussion around periviable birth.

Reviewer comment: Figure 1a: I think this would be easier to follow in a two-column table.
Response: Agreed and Figure updated.

Reviewer comment: Figure 1b: This needs greater explanation- the name of the tool should be described and how the results are to be interpreted- e.g. the chronological age of the child and the corresponding age of the most advanced milestone achieved in each domain?
Response: The following caption has been added to the Figure 1 legend to aid clarity of interpretation for readers: 'Figure 1b illustrates the child's corrected chronological age (grey bar) with the highest achieved, parent-reported milestone from the development tool shown with different coloured bars for each developmental domain.' The name of the tool has been included in the main manuscript text along with the reference for the tool.

Reviewer comment: Figure 1c: I like how this is visually presented
Response: Thank you.

Reviewer comment: Limitations: in the discussion of Figure 2, it is important to acknowledge that there was inherent selection bias in this study - parents of periviable infants who died before two years of age were not included. At the most preterm gestation (22 weeks), a majority of infants delivered will die and this group are not represented here. This is clear in the title of the study. However, it should be mentioned in the discussion of the thematic analysis of "pervasive pessimism" and "balancing hope and realism" as it is only survivors being interviewed, even though they may represent only a minority of cases counselled at periviable gestations.
Response: Agreed – this paragraph has been updated to highlight to the reader that the survey exclusively gathered responses from parents of surviving children. The paragraph now reads as so, "While parents valued honest communication about relevant risks for their baby, many parents experienced these conversations as disproportionately focused on negative outcomes with limited discussion of even the possibility of survival. Although this survey solely represents the views of parents whose child survived, the survey responses showed that parents wanted balanced conversations which acknowledged the inherent uncertainty that exists within periviable care."

VERSION 2 – REVIEW

**REVIEWER NAME**	Murphy, Claire

**REVIEWER AFFILIATION**	Chelsea and Westminster Hospital NHS Foundation Trust, Department of Neonatology
**REVIEWER CONFLICT OF INTEREST**	No Conflict of Interest
**AI DISCLOSURE**	No, I have not used AI
**DATE REVIEW RETURNED**	10-May-2026

**GENERAL COMMENTS**	The authors have addressed my concerns in relation to this article. I recommend it for publication.<br>Minor points<br>Figure 1a- first row should have headings for each column e.g. clinical demographics and numbers
